# Correlating Rheological Properties of a Gellan Gum-Based Bioink: A Study of the Impact of Cell Density

**DOI:** 10.3390/polym14091844

**Published:** 2022-04-30

**Authors:** Annalisa Martorana, Giovanna Pitarresi, Fabio Salvatore Palumbo, Giuseppe Barberi, Calogero Fiorica, Gaetano Giammona

**Affiliations:** Dipartimento di Scienze e Tecnologie Biologiche Chimiche e Farmaceutiche (STEBICEF), Università degli Studi di Palermo, Via Archirafi 32, 90123 Palermo, Italy; annalisa.martorana@unipa.it (A.M.); giovanna.pitarresi@unipa.it (G.P.); fabiosalvatore.palumbo@unipa.it (F.S.P.); giuseppe.barberi01@unipa.it (G.B.); gaetano.giammona@unipa.it (G.G.)

**Keywords:** gellan gum, ionotropic crosslinking, schiff base, cell densities, bioink

## Abstract

Here, for the production of a bioink-based gellan gum, an amino derivative of this polysaccharide was mixed with a mono-functionalized aldehyde polyethyleneglycol in order to improve viscoelastic macroscopic properties and the potential processability by means of bioprinting techniques as confirmed by the printing tests. The dynamic Schiff base linkage between amino and aldehyde groups temporally modulates the rheological properties and allows a reduction of the applied pressure during extrusion followed by the recovery of gellan gum strength. Rheological properties, often related to printing resolution, were extensively investigated confirming pseudoplastic behavior and thermotropic and ionotropic responses. The success of bioprinting is related to different parameters. Among them, cell density must be carefully selected, and in order to quantify their role on printability, murine preostoblastic cells (MC3T3-E1) and human colon tumor cells (HCT-116) were chosen as cell line models. Here, we investigated the effect of their density on the bioink’s rheological properties, showing a more significant difference between cell densities for MC3T3-E1 compared to HCT-116. The results suggest the necessity of not neglecting this aspect and carrying out preliminary studies to choose the best cell densities to have the maximum viability and consequently to set the printing parameters.

## 1. Introduction

Traditional methods of obtaining scaffolds usually fail to fabricate a realistic 3D physiological microenvironment [1]. The seeding of cells on the surface of a previously obtained scaffold represents an inefficient strategy due to the uncontrollable and non-uniform deposition of cells on the entire matrix [2]. However, 3D bioprinting, an additive manufacturing technology that works by layer-by-layer deposition, enables accurate spatial control during fabrication of constructs biomaterial/cells. Such highly customized 3D constructs can be employed for tissue engineering purposes or for production of complex models for in vitro tumor and drug screening studies [3,4,5]. Extrusion-based technology represents the most common biofabrication method due to its relative simplicity, low cost, minimal cell damage and versatility [6,7,8]. Hydrogels are often used as inks, and polysaccharides have been considered attractive vehicles for encapsulating and releasing cells due to their ability to reproduce the stiffness, porosity and physical properties of the native extracellular matrix (ECM) [9,10,11,12]. However, there are several challenging requirements and design considerations in the development of a new hydrogel-based bioink [13,14,15]. The major effort in this direction consists in improving the printability, selecting all the parameters to obtain the best construct and to ensure that all the applied strategies do not reduce cell viability [16,17,18]. Appropriate viscosity, ability to acquire adequate mechanical stabilization after extrusion, gel fracture, use of a cross-linking agent, high temperatures and high stresses applied to the nozzle are key determinants in the bioprinting process affecting the viability of encapsulated cells [16,19,20,21]. Furthermore, crosslinking methods play a crucial role in cell printing and response [22,23] and, due to the intrinsic dynamic equilibrium of the bond in a Schiff base [24,25], recently pseudo-covalent links have been used successfully for 3D printing purposes [26,27,28,29,30,31]. To further complicate the matter, the cell types and hydrogel used can influence the magnitude of these effects and the rheological properties of the bioink [32]. Many efforts have been made to investigate the relationship between cell viability [8,16,33,34,35] and nozzle tip type, diameters [36], printing speed and applied pressure [37], while the influence of different cell lines and density on printing parameters has often been ignored [33,38]. Cell density is essential to facilitating initial cell–cell interactions towards tissue maturation [39,40,41].

Typically, to successfully perform 3D bioprinting, chemical modified polymers have been used or more biomaterials have been combined to match different properties [12,42]. The introduction of new substituents on the backbone of natural polysaccharides, such as the –NH_2_ group, can not only allow the modification of the physical properties of these biocompatible polymers, producing new materials for different applications, but can also open new further developments [43].

Gellan gum (GG), an anionic polysaccharide, is a natural biopolymer with important features for the tissue engineering field [44,45], which has already been modified to be used as a bioink [46,47]. 

The responsiveness of GG to external medium ionic strength is one of the most representative features of this polysaccharide, and the ionotropic gelation with mono or divalent cations has been exploited to produce physical hydrogels for tissue engineering and drug delivery purposes [48,49]. Unfortunately, the brittleness and weak mechanical properties of crosslinked ionotropic GG hydrogels have made it necessary to perform chemical modifications on the polysaccharide backbone or to use polymer blends to obtain printable dispersions and stable hydrogels [47,50,51,52]. For example, Jongprasitkul et al. [53] demonstrated that it is possible to use GG methacrylate to obtain a printable ink that can be crosslinked with a combined ionotropic and photoinduced crosslinking while Wu et al. [54] combined the GG dispersion with poly (ethylene glycol) diacrylate to obtain printable inks with superior rheological properties, printability and fast UV curing capabilities. 

In our previous work, we proposed an ethylenediamino functionalized GG derivative, called GG-EDA, synthesized by using a low molecular weight GG, in order to obtain a polyampholite that forms hydrogels with improved stability and an enhanced elasticity (compared to starting GG with similar molecular weight) due to the physical interactions of inserted amine groups with glucuronic moieties of tetrasaccharide repetitive units. 

In this work, we propose to use GG-EDA as a starting biomaterial to produce a bioink by mixing it with aldehyde mono functionalized polyethylene glycol. Our general idea was to exploit the dynamic Schiff base linkage between amino and aldehyde groups to modulate the initial viscosity of the systems and obtain a crosslinkable ionotropic ink capable of recovering the initial mechanical properties due to the physical interactions of GG- EDA.

In fact, it has been shown that the mixing of the two polymers determines an improvement of the viscoelastic macroscopic properties and the potential processability by means of bioprinting techniques.

For this reason, in order to study the influence of cell encapsulation of the newly designed bioinks, we selected human colon cancer and preosteblastic cell lines by studying how rheological properties depend on them. Particularly, we investigated from a rheological point of view the influence of three densities of these two cell lines into the gellan gum-PEG-based hydrogels with the aim of evaluating their possible impact on the printing process. 

## 2. Materials and Methods

### 2.1. Materials

Gellan Gum (Gelzan^TM^ CM) of low acyl degree, sodium hydroxide (NaOH), ethylendiamine (EDA), tetrabuthylammonium hydroxide (TBA-OH), bis(4-nitrophenyl)carbonate (4-NPBC), acetone, dimethylsulfoxide anhydrous (DMSO_a_), Dowex^®^ 50WX8 hydrogen form, O-[2-(6-Oxocaproylamino)ethyl]-O′-methylpolyethylene glycol 2000 (PEG-aldehyde 2000), Live/dead staining kit, Dulbecco’s phosphate buffered saline (DPBS), and Dulbecco’s Minimum Essential Medium (DMEM) deuterium oxide (D_2_O) were purchased from Sigma-Aldrich (Milan, Italy). Hydrochloric acid (HCl) was purchased from Fluka (Milan, Italy).

CellTiter 96^®^ AQueous One Solution Cell Proliferation Assay (MTS) was purchased from Promega (Milan, Italy). HCT-116 (human colon tumor) and MC3T3-E1 (murine preostoblastic cells) were purchased from Euroclone (Milan, Italy).

### 2.2. Apparatus

^1^H-NMR spectra were obtained with a Bruker Avance II 400 MHz spectrometer (Milan, Italy).

FT-IR spectra were obtained with Bruker Alpha in the wave number range of 400 and 4000 cm^−1^ (Milan, Italy). SEM analyses were performed with Phenom Pro X Desktop (Thermo Fisher Scientific, Rome, Italy).

The rheological tests were carried out using a DHR-2 TA Instrument rotational rheometer (TA Instruments-Waters S.p.A., Sesto San Giovanni, Italy).

UV measurements were performed using an Eppendorf AF2200 spectrophotometer (Milan, Italy).

Cell cultures were performed using an Eppendorf New Brunswik S41i incubator (Milan, Italy).

Fluorescence images were obtained with an AxioVert200 (Zeiss) microscope (Milan, Italy). 

A 3D printer Zmorph (Wroclaw, Poland) connected with a pressure controller OB1 working at pressures of 0–8000 mbar from Elveflow (Paris, France) was used to test printability of the inks. 

### 2.3. Preparation of GG-EDA-PEG Inks and Characterization

Gellan gum-((2-aminoethyl)-carbamate) (GG-EDA) was produced as previously reported [55] and the functionalization degree in EDA moieties was equal to 40 ± 5 mol% calculated by ^1^H-NMR analysis and 2,4,6-trinitrobenzene sulfonic acid (TNBS) assay.

GG-EDA was dispersed in bidistilled water at a concentration of 1% *w*/*v* and kept at 90 °C for 1 h to obtain a complete and homogeneous dispersion. Subsequently, the pH was adjusted to pH 6.5 by adding HCl 1N, and then the solution was freeze-dried. 

GG-EDA-PEG inks were prepared simply by mixing a PEG-Ald 2000 water solution (3% *w*/*v*) with a GG-EDA aqueous hot (80 °C) dispersion (3% *w*/*v*) under vortex agitation. Two different molar ratios X’ e X’’ of PEG-Ald and amine groups of GG-EDA, equal to 0.2 and 0.4, respectively, were used to obtain two different inks called GG-EDA-PEG A (ink A) for the molar ratio 0.2 and GG-EDA-PEG B (ink B) for the molar ratio 0.4. For both inks, the final polymer concentration was set at 3% *w*/*v*. ^1^H-NMR and ATR-FTIR analyses were performed on freeze dried GG-EDA-PEG based inks.

Freeze dried samples were characterized by Phenom Pro X Desktop Scanning electron microscopy (SEM).

### 2.4. Stability of GG-EDA-PEG Derived Hydrogels

GG-EDA-PEG hydrogels were produced from freeze dried inks by ionic crosslinking with DPBS pH 7.4. For stability studies, samples were weighed (W_i_) and placed into a 24 well cell culture plate. Each sample was submerged with DPBS pH 7.4 (2 mL), and the plate was incubated in an orbital shaker incubator at 37 °C. 

At determinate times (7, 14, and 21 days), samples were gently washed with bidistilled water to remove DPBS salts and then freeze-dried and weighed.

Recovered weight % (*W_r_%*) was calculated using the following equation:

(1) $$W_{r}\%=\frac{W_{f}}{W_{i}}\times 100$$

where *W_f_* represents the recovered weight at the selected time, while *W_i_* represents the initial weight. Each experiment was performed in triplicate.

^1^H-NMR analyses were performed on samples incubated for 7 days, after washing and freeze-drying.

### 2.5. Rheological Characterization of GG-EDA-PEG Based Inks

To perform rheological measurements, hydrogels were prepared as disks. In particular, 200 µL of aqueous dispersion of 3% *w*/*v* of GG-EDA-PEG A or B, obtained as described before, was added on the plates of a 48-well cell culture plate, and these circular samples have been used for rheological analyses. 

Oscillation amplitude tests were conducted by applying a strain percent in a range between 0.1% and 1000%, and a linear sweep angular frequency at 1.0 rad/s at 25 °C to measure the storage modulus (G′) and loss modulus (G′′). 

Flow sweep experiments were conducted at 25 °C for all samples to measure viscosity by changing the shear rate (0.01 to final 10^2^ s^−1^) for 60 s. 

Thermo-rheological experiments were conducted from 60 to 5 °C at a rate of 2 °C/min by applying a constant strain of 1% in the linear viscoelastic region and a frequency of 1 rad/s. Frequency sweep measurements were performed at a constant temperature (25 °C) over a range of oscillation frequencies between 0.06283 and 62.83 rad/s, applying a constant strain of 1%. Recovery studies were performed at 25 °C and with an angular frequency of 1 rad/s, applying a constant strain of 1% for 100 s followed by a constant strain of 500% for 100 s for a total of seven steps.

The measurement gap was set at 300 µm for all analyses and the excess sample was carefully removed before starting the experiment. All experiments were carried out in triplicate. 

Moreover, frequency sweep measurements were performed, under the same experimental conditions, on the same samples incubated for 1 h in DMEM.

### 2.6. 3D Printing

Printing tests were performed with Zmorph connected with an OB1 pressure controller. 

GG-EDA-PEG based inks were prepared as described above, and thymol blue at a concentration of 0.1% *w*/*v* was added for better visibility of the printed structure. The shape chosen was a honeycomb structure with a diameter of 2 cm, produced with the Voxelizer software.

The extrusion process was performed with a 27 G (0.413 mm) needle, the air pressure was 1200 mbar and the print speed of 14 mm/s for the ink A, while the air pressure was 500 mbar and the print speed of 20 mm/s for the ink B.

The shape fidelity was calculated by measuring the area of the hexagonal mesh of the printed structure and the area of the hexagonal mesh of the digital model [54].

(2) $$F_{ma}=\frac{A_{e}}{A_{t}}\times 100$$

where *F_ma_* is the parameter called fidelity on mesh area which is an index of the printability and resolution of the construct compared to the digital model and *A_e_* is the experimental mesh area calculated on the printed structure, while *A_t_* is the theoretical area calculated on the digital model. The measurements were performed with the Imagej software.

### 2.7. Bioink Preparation, Rheological Characterization and In Vitro Cytocompatibility Studies

Freeze dried GG-EDA was sterilized by UV irradiation for at least 30 min, using a 125 W UV lamp working at 254 nm, and dispersed in sterile water. PEG dispersion was sterilized by filtration using a cellulose acetate 0.2 µm filter (Whatman, Milan, Italy).

For bioink preparation, inks A and B were prepared as previously described but with a final concentration equal to 3.3%. These solutions were diluted to a concentration of 3% with the same volume of cell dispersion containing a different number of cells. MC3T3-E1 e HCT-116 were cultured in DMEM, which was prepared with 10% *v*/*v* fetal bovine serum, 1% *v*/*v* penicillin/streptomycin, 1% *v*/*v* glutamine and 1% *v*/*v* amphotericin B. Cells were cultured in T75 flasks that were fed every two days and incubated at 37 °C under a 5% CO_2_ atmosphere.

After trypsinization, cells were suspended in an equal volume of DMEM and added to a dispersion of GG-EDA-PEG A or B to have a final concentration of 3%. As a control, the same volume of DMEM was added to the polymer dispersions without cells. 

Immediately after preparation, rheological measurements, ionotropic crosslinking and in vitro cytocompatibility studies were performed. 

In particular, rheological experiments were performed at 25 °C for the selected cell densities 1 × 10^7^, 1 × 10^6^ and 1 × 10^4^ cells/mL. To ensure that the cells were evenly distributed, the solutions were kept in a water bath (37 °C) and each sample was gently shaken before pipetting the sample onto the rheometer plate. Before each test, the samples were cooled to room temperature through the temperature control unit in the rheometer.

Oscillation amplitude and recovery time studies were conducted in the same manner for inks without cells. For each test, 200 µL of the sample was pipetted onto a 48-well cell culture plate and left at 25 °C before use. Further characterization with frequency sweep measurements was performed to evaluate the mechanical performance and the effect of ionotropic crosslinking (cured samples). For the ionotropic crosslinking, 200 µL of the samples was pipetted in DMEM (1 mL) into a 48-well cell culture plate. After 7 days of incubation at 37 °C under a 5% CO_2_ atmosphere with the medium refreshed every 2 days, frequency sweep analyses were conducted at (37 °C), in the range between 0.06283 and 62.83 rad/s (0.010–10 Hz), strain % 1.0%. 

In vitro cytocompatibility studies were performed at 1 and 7 days with MTS and Live&Dead assays. For MTS, 100 µL of each sample was pipetted with a 27 G needle in DMEM (1 mL) onto a 48-well cell culture plate.

Then, absorbance was measured at 492 nm. Analyses were performed in triplicate. For Live&Dead assays, 100 µL of each bioink was injected with the same needle onto a 2-well Nunc chamber slide, as filaments. Then, the samples were incubated with DMEM (500 µL) and at determined time (1 and 7 days), Live&Dead assay was performed. The bioink solutions were extruded with a 1 mL syringe with a 27 G needle into a cover glass container. A Live/Dead staining kit was prepared following the standard protocol (PromoKine Live/Dead Staining Kit II, Heidelberg, Germany). After 30 min of incubation, images were taken using fluorescence microscopy. Images were processed with ImageJ (FIJI) software.

At the time of determination, the DMEM was removed, and the samples were washed gently with DPBS, after adding 500 µL of Live & Dead solution to each well containing the sample, and the plate was incubated for 30 min at 37 °C; the images were taken with an AxioVert200 fluorescence microscope (Zeiss).

### 2.8. Statistical Analysis

All results are reported with a media ± standard deviation and, when applicable, the statistical analysis for significance was conducted with the Student’s *t*-test, utilizing the function *t*-test of Microsoft Excel, assuming the non-homogenous variance at 2 samples and a dual tile distribution; values with *p* < 0.05 were considered statistically significant.

## 3. Results and Discussion

### 3.1. Preparation of GG-EDA-PEG Inks and Characterization

The development of new biomaterials for bioink production is a fascinating research field since, despite the innumerable advantages brought by the 3D printing biofabrication technique compared to other scaffold production techniques, the bioinks used are relatively few and most of them are produced with the same starting macromolecules (first of all, gelatin methacrylate).

In order to obtain new inks with appropriate characteristics to improve the performance with a 3D printer we propose to use, as a starting material, GG-EDA. Introducing free amine groups on the gellan gum backbone allows further functionalization [56] with other molecules capable of influencing the physicochemical and biological properties, increasing cell adhesion [57]. Furthermore, this functionalization does not modify the stimulus-sensitive properties of the polysaccharide, such as the sensitivity to the ionic strength of the medium, thereby allowing researchers to obtain a derivative that still undergoes an ionotropic gelation with mono and divalent cations.

Moreover, this derivative is characterized by inter- and intra-molecular interactions between the amine group of the EDA portion and the carboxyl group of the gellan gum. Although a low molecular weight gellan gum was used, because of these interactions, aqueous dispersion of GG-EDA showed a higher viscosity value than starting gellan gum. Despite the fact that this new feature is helpful for maintaining the shape after the deposition of the material during a 3D printing process [21], without the need for other, usually cytotoxic, crosslinking methods [58], the remarkable dispersion viscosity, at a concentration useful for obtaining crosslinked ionotropic structured hydrogel, does not allow the cells to be safely and homogeneously encapsulated, further reducing injectability.

Therefore, to modulate the viscoelastic properties of the gel forming system and improve the extrusion process, the formation of a dynamic linkage was exploited. 

**Figure 1:**
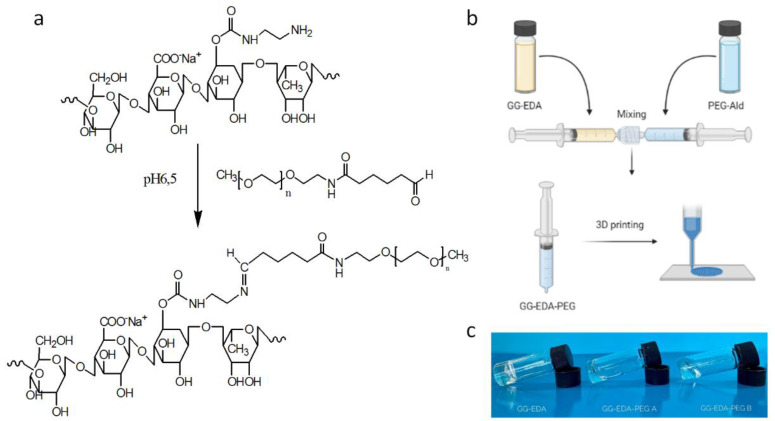
(**a**) Schematic representation of GG-EDA-PEG synthesis and (**b**) preparation of inks; (**c**) Inversion tube test of GG-EDA, GG-EDA-PEG A and GG-EDA-PEG B.

Considering the characteristics of PEG, such as the absence of toxicity, non-immunogenicity and biocompatibility [59,60,61], we used PEG-Ald 2000 to react with GG-EDA by means of a basic Schiff reaction, to reduce the transition temperature and viscosity and thus facilitate mixing with the cell dispersion and obtain a printable system (Figure 1a,b).

Although PEG was already used to graft aminated polymers such as chitosan [62], reduction of linkage amination has usually been reported [63,64]. In this case, PEG was used as sacrificial material. The sacrificial function of the PEG, given by the intrinsic imine linkage, temporarily reduces the intra- and inter-molecular interactions of GG-EDA described above to print the bioink and allow the recovery of the original properties of the gellan gum in vivo over time, also thanks to the ionotropic-induced gelation of the polysaccharide. In this way, a polymeric dispersion is easily extrudable, without applying high pressures, and exploitable, for cell encapsulation can be obtained. Finally, we were able to print the obtained biomaterial, called GG-EDA-PEG, and at the same time, we prolonged the degradation profile of the polysaccharide thanks to the physical interactions that strongly contribute to the mechanical properties. 

In particular, to study the influence of PEG, two different molar ratios were considered to obtain the inks, A (20 mol% with respect to the GG-EDA amine groups) and B (40 mol% with respect to the GG-EDA amine groups). 

The obtained inks have shown different macroscopic characteristics of fluidity compared to GG-EDA at the same concentration, as shown in the Figure 1c with the inversion tube test. As expected, the dispersion viscosity decreases as the amount of PEG increases. 

**Figure 2:**
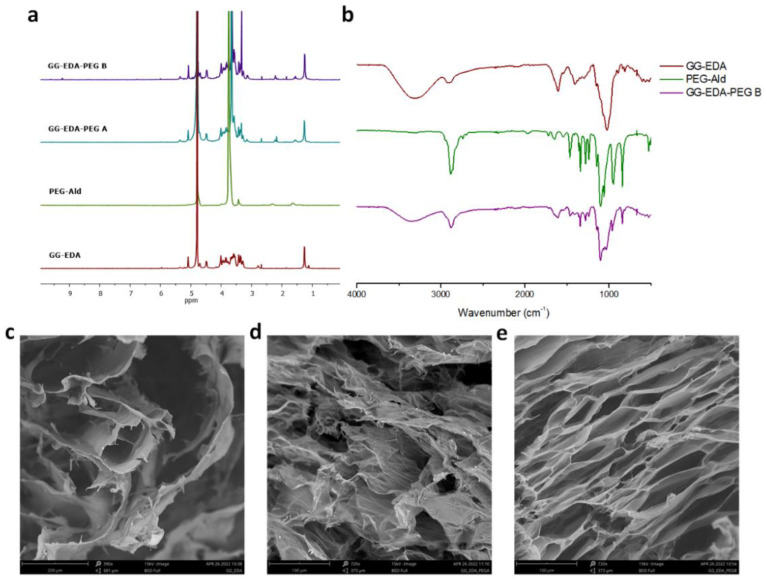
(**a**) ^1^H-NMR spectra of GG-EDA, PEG-Ald, GG-EDA-PEG A and B; (**b**) ATR-FTIR spectra of GG-EDA, PEG-Ald, GG-EDA-PEG B; SEM images of (**c**) GG-EDA, (**d**) GG-EDA-PEG A and (**e**) GG-EDA-PEG B.

^1^H-NMR spectra (Figure 2a) showed an intense peak at δ 3.6 relative to C**H_2_**, δ 1.55 (NHCOCH_2_C**H_2_**C**H_2_**), δ 2.15 NHCOC**H_2_** of PEG and the peak at δ 1.2 C**H_3_** relative to the rhamnose of the gellan gum for both samples, GG-EDA-PEG A and B. 

ATR-FTIR analysis of GG-EDA-PEG inks was performed on freeze-dried samples. For simplicity, GG-EDA-PEG B spectra were reported. The GG-EDA-PEG B sample showed the typical PEG peaks (2876 cm^−1^ and 1102 cm^−1^ compared to CH stretch and CO stretch, respectively, and 1280, 947 and 843 cm^−1^) with significantly reduced intensities compared to PEG alone as a result of PEG bound to GG-EDA [65,66]. Furthermore, the peak at 1721 cm^−1^ (CHO), related to the PEG-Ald aldehyde group disappeared as a consequence of the linkage formation [67]. In fact, the GG-EDA-PEG B spectra show a peak at 1650 cm^−1^ probably associated with imine binding (Figure 2b).

SEM images of GG-EDA, GG-EDA-PEG A and GG-EDA-PEG B are reported in Figure 2c–e, respectively. The presence of PEG in both inks is reflected in a denser mesh structure compared to GG-EDA. This may be due to PEG which, interposing between GG-EDA chains, allows a better solubility of the latter, thereby resulting in a smaller mesh size.

### 3.2. Rheological Characterization of the Inks

**Figure 3:**
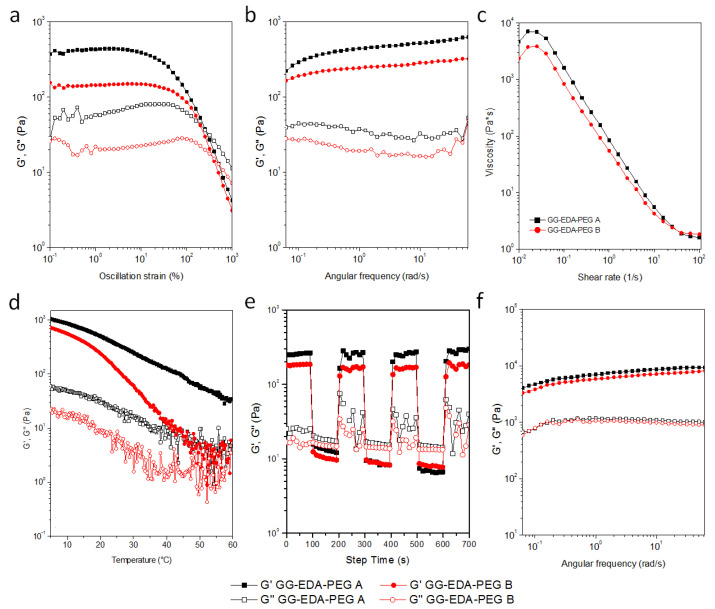
Results of rheological analyses of (**a**) oscillation amplitude, (**b**) frequency sweep, (**c**) flow sweep, (**d**) temperature sweep and (**e**) recovery time; (**f**) Results of frequency sweep measurements of one hour incubated samples in DMEM.

Rheological characterization can provide insight into the 3D printing process, impact on cell viability, and material recovery after extrusion (Figure 3). Measurements of the amplitude of the oscillation (Figure 3a) were performed to study the behavior of the G′ and G′′ moduli over a wide strain range. The linear viscoelastic region (LVR, at low% strain values) and the crossover of the G′ and G′′ (at higher % strain values) were determined.

Both samples show gel-like behavior, with differences of approximately one order of magnitude between G′ and G′′, with G′ value of ink A greater than ink B.

With regard to G′, the two inks have a different LVR (up to about 5% and 15% of strain for ink A and ink B, respectively). As the % of deformation increases, G ′ rapidly decreases with a crossover of moduli for ink A and B at 250% and 360% of deformation, respectively, as a direct consequence of the greater structuring of ink A as expected due to the lower amount of PEG-Ald.

Frequency sweep analyses showed G′ and G′′ almost independent of the tested frequency range with higher moduli values for 220 to 620 Pa ink A compared to 160 to 320 Pa ink B (Figure 3b).

The pseudoplastic behavior was confirmed by flow sweep measurements; both samples showed a reduction in viscosity with increasing shear rate (Figure 3c). In particular, the viscosity value of ink A at a low shear rate was higher than ink B (7000 and 3800 Pa × s respectively at 0.015 s^−1^) due to more EDA-free groups in early samples providing more structured hydrogels.

As the applied shear rate increases, the viscosity values decrease for both samples, which thus reach similar values.

Temperature strongly influences the viscosity and moduli values. For this reason, temperature sweep analyses were performed in the range of 60–5 °C and results are reported in Figure 3d. Gellan gum can pass from a coil to the helix structure as the temperature decreases with an increase in the moduli values. In particular, the transition strongly depends on molecular weight and concentration [45] and both moduli are inversely related to temperature. It is interesting to note that ink B, containing a greater quantity of PEG, showed a variation in behavior G′ similar to that of non-functionalized (sigmoidal) gellan gum while, as the temperature decreased, the increase of modulus of the ink A was more gradual. Probably a lower quantity of PEG causes a lower perturbation of the chains. The PEG chains, in fact, disturb the entanglement of the GG-EDA macromolecules, interposing between them and, probably, blocking part of the amino groups, resulting in better fluidity and causing the gel-sol transition to occur earlier. Finally, the recovery test analyses were performed by applying the 7-cycle deformation test performed as oscillation time scans under alternating step strain% as shown in Figure 3e. For both polymers, at high deformation, an inversion of the moduli is obtained, and when the deformation was established at 1%, good recovery was shown with a reduction of G′ from 370 to 20 Pa for ink A and from 200 Pa to 10 Pa for B ink. This behavior has been confirmed for all steps. 

Frequency scan measurements were also performed on the samples of the two inks incubated in DMEM for one hour (Figure 3f).

Frequency sweep measurements on the samples of the two inks incubated in DMEM for one hour (Figure 3f) were also performed. As can be seen, there is a notable increase, of an order of magnitude, for the moduli of both inks, and the differences between the two samples decrease considerably (for the inks A G’ range is 4000–9300 Pa, while for the inks B is 3300–8200 Pa). 

This allows us to state that both inks maintain the sensitivity to the ionic strength of the medium by reducing the differences between the two samples in terms of viscoelastic properties.

### 3.3. Stability of GG-EDA-PEG Derived Hydrogels

**Figure 4:**
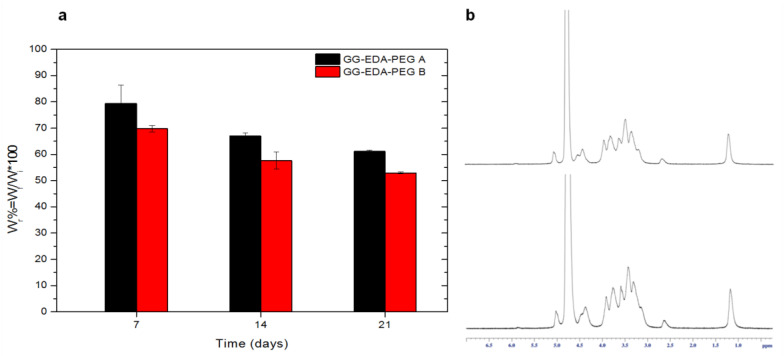
(**a**) Results of the degradation tests: percentage of recovered weight (W_r_) as a function of time, of the scaffolds of the two polymers GG-EDA-PEG A and B at 37 °C in DPBS; (**b**) ^1^H-NMR spectra of GG-EDA-PEG A (above) and B (below) after 7 days of degradation.

Stability studies revealed, for the hydrogels obtained by crosslinking with DPBS pH 7.4 of ink A and ink B, a weight loss after 7 days of incubation of 20 ± 6.95% and 30 ± 1.24%, respectively (Figure 4a). This weight loss corresponds to the amount of PEG in the inks and is ascribable to the elimination of the synthetic polymer from the hydrogels. To confirm this hypothesis, samples were recovered, freeze-dried and a ^1^H-NMR analysis was performed. As shown in Figure 4b, the peak at δ 3.6 attributable to -CH_2_ of PEG is not observable.

This result corroborates the rationale of using PEG as a sacrificial polymer in the ink.

On the 14th and 21st days, the weight loss is much less than on the 7th day, due to the high hydrolytic resistance of GG-EDA.

### 3.4. 3D Printing

After the development and characterization of the inks, preliminary tests were carried out to study the behavior in the extrusion phase and verify the effective printability and resolution of the constructs obtained.

**Figure 5:**
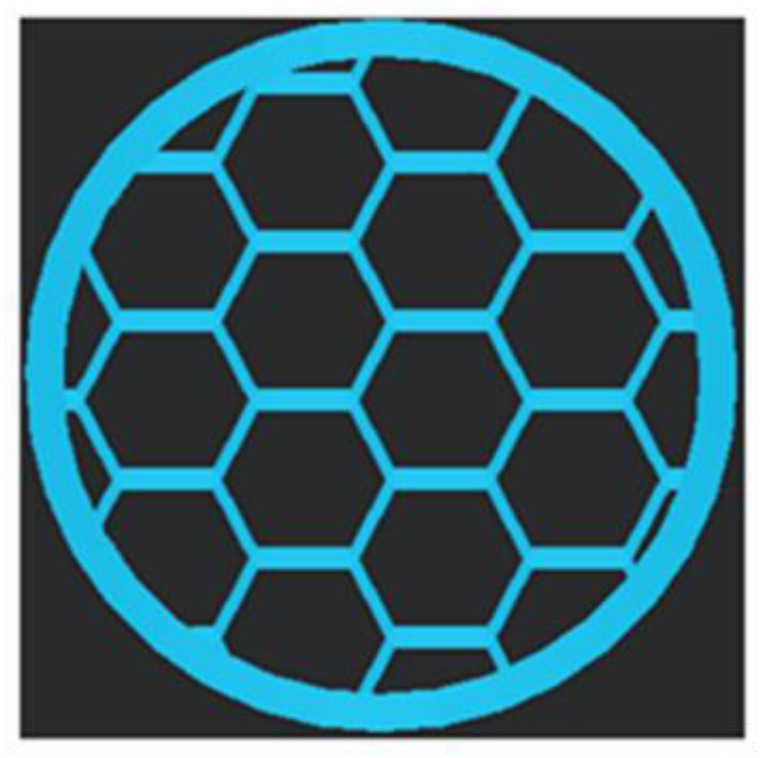
Honeycomb structure, with a diameter of 2 cm, used as a digital model for printing tests.

The tests were carried out using a Zmorph printer connected to a pressure control system; the digital model shown in Figure 5 was produced with the Voxelizer software (Version number 2, created by ZMorph, Wroclaw, Poland). 

It is known that the type of material used, the pressure exerted, the printing speed and, above all, the size of the nozzle greatly influence the resolution of the printed construct [16,21]. Consequently, in order to identify the optimal setting, several tests were performed that varied different parameters, such as pressure and extrusion speed.

To make the printed structures more visible and to study their compliance with the digital model produced, thymol blue (0.1% *w*/*v*) was added to the dispersions of both inks.

**Figure 6:**
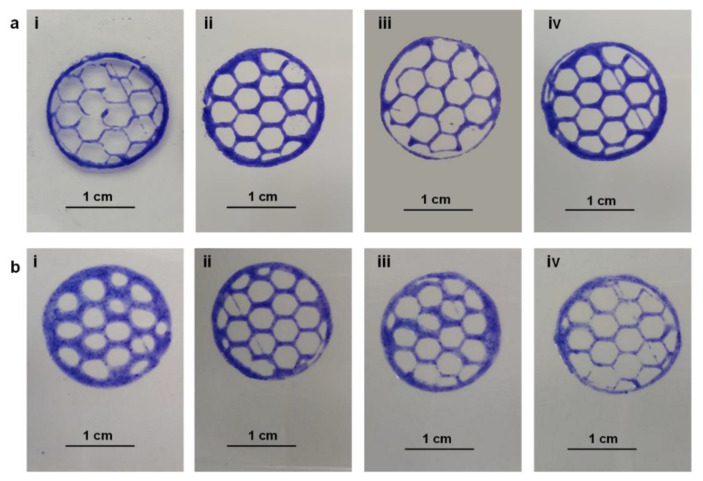
Printing test results for inks (**a**) A and (**b**) B. Each reported image was obtained under different pressure and printing speed conditions: (**a**) 1000 mbar and 20 mm s (**i**), 1200 mbar and 14 mm/s (**ii**), 1000 mbar and 22 mm/s (**iii**), 1200 mbar and 16 mm/s (**iv**); (**b**) 400 mbar and 22 mm/s (**i**), 500 mbar and 20 mm/s (**ii**), 500 mbar and 24 mm/s (**iii**), 500 mbar and 25 mm/s (**iv**).

Figure 6 shows the results obtained with inks A and B, respectively, at different pressure and speed conditions using a needle with a diameter of 27 G (0.413 mm).

For ink A, pressure and print speed in the range 1000–1200 mbar and 14–22 mm/s have been tested. The best resolution was achieved with a print pressure of 1200 mbar and with the lowest print speed of 14 mm/s (Figure 6a(ii)).

For ink B, on the other hand, pressure and print speed were tested in the range 400–500 mbar and 20–25 mm/s. The best resolution was achieved with a print pressure of 500 mbar and a speed of 14 mm/s (Figure 6b(ii)).

As can be seen, ink A, having values of G′, G′′ and higher viscosity than ink B, requires higher pressure values and lower printing speed than ink B.

Furthermore, to evaluate the printing precision, the area of the hexagonal meshes present in the printed structures was calculated and compared with that of the digital model.

These measurements were carried out with the Imagej software. The percentage ratio was about 85.70 ± 4.95% for ink A and 81.20 ± 5.45% for ink B. In addition, the thickness of the fibers was measured and correlated with the diameter of the needle used for the printing procedure. It was observed that, for both inks, the fiber thickness/needle diameter ratio was 1.41 ± 0.09%. All this demonstrates that, under different conditions of pressure and printing speed, both inks have good conformity to the expected pattern and can be effectively used in 3D bioprinting applications. However, the lower pressure required for the extrusion of ink B means that less pressure is exerted on possibly encapsulated cells, resulting in less damage. This aspect would make ink A preferable.

### 3.5. Bioinks Preparation and Rheological, Ionotropic Crosslinking and In Vitro Cytocompatibility Studies

The design of innovative bioinks with optimized parameters tailored to a specific type of tissue is one of the main objectives of 3D bioprinting.

Some of the factors affecting bioink properties are hydrogel concentration, viscosity, cellular density and the crosslinking method. An appropriate bioink with adequate structural and mechanical properties should protect cells from damage during printing and direct or control their phenotype and function by simulating an appropriate physiological environment [68]. Usually, different cell types respond differently to the microenvironment; therefore, depending on these aspects, the properties of the bioink should be regulated. For example, the concentration of hydrogel or the solvent used to prepare the bioink strongly affects cell viability [34,69,70]. Furthermore, the cell type may respond differently to temperature and shear stress, which require additional attention to set the parameters [16].

Here, considering the different context in which bioprinting can act, HCT-116 and MC3T3-E1 cells were selected to test how different cells influence the rheological properties of the bioink.

The concentration of the polymer was maintained at 3% *w*/*v* and three different cell densities were selected (10^4^, 10^6^, and 10^7^, cells/mL, respectively, called 10 K/mL and 1 and 10 M/mL) to study how the cells loaded into the biomaterial can affect both rheological processing and post-print cell viability for tissue growth [18,71,72].

The characterization of bioinks consists of rheological analyses, ionotropic crosslinking and in vitro cytocompatibility studies.

#### 3.5.1. Rheological Characterization of the Bioinks

Billiet et al. fabricated scaffolds loaded with cells by testing the influence of cell density by encapsulating the hepatocellular carcinoma cell line (HepG2) and demonstrating an inverse correlation between cell numbers (ranging from 0.5 × 10^6^ to 2.5 × 10^6^ cells/mL) and viscosity [72].

Here, we performed rheological measurements of bioinks in order to study the effect of cell density on injectability and recovery in the extrusion process.

**Figure 7:**
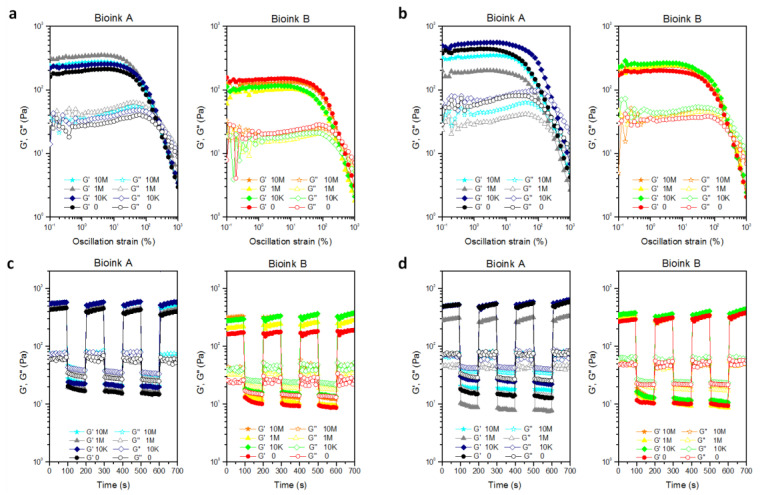
Results of (**a**,**b**) oscillation amplitude and (**c**,**d**) recovery time on bioinks A and B encapsulating (**a**,**c**) HCT-116 and (**b**,**d**) MC3T3-E1 at different densities.

Different behaviors of moduli are shown from strain % spectra depending on the cell line and the considered bioink (Figure 7a,b). In particular, a different impact with important effects has been reported for bioink A compared to bioink B, whose modules are not significantly affected by cell density as the strain % increases. Furthermore, the effect of cell density increases depending on the cell type with differences enhanced for MC3T3 compared to HCT-116 cells. In fact, for bioink A, it was observed that, for all the cell densities tested, the presence of HCT-116 determines an increase in the viscoelastic modulus compared to the cell-free sample, even if there is no direct correlation between the increase in G′ modulus and cell density.

In particular, the bioink showing a higher G′ value was the one with a cell density of 1 M/mL, while the bioink with a cell density of 10 M/mL and the one with 10 K/mL showed G′ values that were comparable and lower than that of the bioink with intermediate cell density (Figure 7a). It is not trivial to consider cells as “microstructures” which, depending on their density in the polymer dispersion, can, on the one hand, contribute to conferring structural properties to the hydrogel, but on the other hand, at higher densities, can perturb the macromolecular entanglements that reduce interactions between polymer chains. With this in mind, it is possible to state that for bioink A, 1 M/mL represents the most suitable cell density for HCT-116. 

Results obtained for bioink A incorporating MC3T3-E1 cells corroborate the “structuring/destructuring” hypothesis. In fact, compared to the cell-free sample, only the density of 10 K/mL (the lowest) determined a slight increase in G′ value compared with the cell-free sample, while the opposite effect was registered with higher cell densities (1 M/mL and 10 M/mL). Probably the larger diameter of suspended MC3T3-E1 cells has a more pronounced destructuring effect on polymeric entanglement than HCT-116. Indeed, immediately after trypsin treatment, cellular suspensions of HCT-116 are about 8 µm smaller in diameter than MC3T3-E1. This correlation between cell density and G′ modulus was not observed for bioink B, with slight variations with respect to the control. In this case, probably the cells, in a structure in which the entanglement is already compromised by the PEG chains, do not have a relevant impact. Studies on recovery times showed that, although with variations in G′ and G″ compared to cell-free hydrogels, none of the cell densities affected the recovery of viscoelastic moduli through low–high deformation cycles (Figure 7c,d).

#### 3.5.2. Ionotropic Crosslinking of Bioinks

As previously discussed, gellan gum-based bioinks in contact with media containing mono and/or divalent cations undergo ionotropic crosslinking, which can contribute to obtaining more structured hydrogels after the printing process—for example, once the construct is implanted in vivo [44].

**Figure 8:**
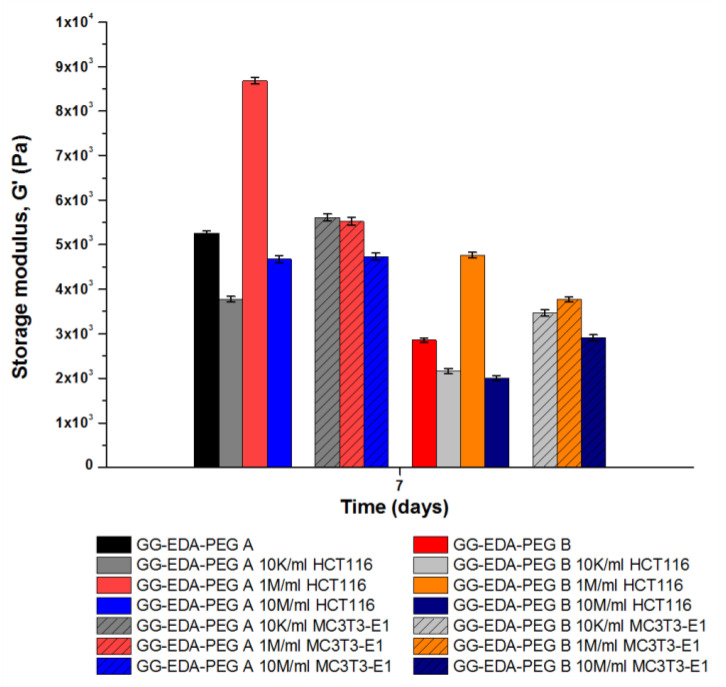
Results of frequency sweep analysis after 7 days incubation in DMEM of bioink A and B encapsulating HCT-116 and MC3T3-E1 at different densities.

Therefore, the aforementioned bioinks were injected into the cell medium (DMEM) for 7 days in order to study the crosslinking effect on the viscoelastic properties of the obtained hydrogels by means of frequency sweep analyses. The results are shown in Figure 8.

Notably, the increased G′ values compared to the non-crosslinked samples confirmed that the presence of cations caused greater interaction between the gellan gum chains for both cell-laden and non-cell-laden samples.

Regarding the effect of cell densities on G′ values, again, different results were obtained depending on the bioink and the cell type selected. When 1 M/mL of HCT-116 were encapsulated on both ink A and ink B, G′ increased and cells encouraged greater interaction between gellan gum chains. Since the cells are micrometric in size, introducing such a number of “particles” into the bioink could further structure it, increasing the G ′ value, without significantly interfering with the ionotropic crosslinking process. However, when cell density was lower (10 K/mL) or higher (10 M/mL), a decrease in G′ values was observed in agreement with what was already observed with oscillation amplitude experiments conducted on non-crosslinked bioinks. On the other hand, regarding MC3T3-E1, there are no significant variations for any cell densities tested for both GG-EDA-PEG A and GG-EDA-PEG B. This may be related to the perturbation of PEG chains, which reduce the ionic crosslinking effect. Moreover, considering the same concentration of both bioinks, as the quantity of PEG increases, a reduction of gellan gum chains, which can undergo the crosslinking process, occurs. 

#### 3.5.3. In Vitro Cytocompatibility Studies

In order to assess the cell viability after the preparation and mixing process with polymers, in vitro cytocompatibility studies were conducted. In particular, bioinks, prepared as reported above, were injected into DMEM and analyzed after 1 and 7 days.

**Figure 9:**
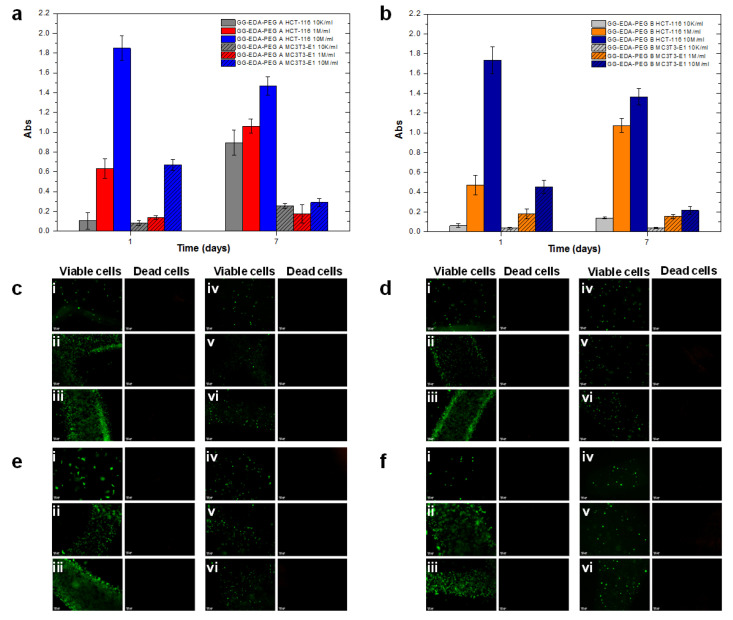
Results of the MTS assay at two different times (1 and 7 days) for the encapsulated HCT-116 and MC3T3-E1 cells, at different densities, within bioinks (**a**) A and (**b**) B. The results are expressed as absorbance values at 490 nm. Results of the L&D assay at (**c**,**d**) 24 h and (**e**,**f**) 7 days for HCT-116 and MC3T3-E1 cells encapsulated, at different densities, within bioinks (**c**,**e**) A and (**d**,**f**) B. The different cell densities were (**i**) 10 K/mL, (**ii**) 1 M/mL, (**iii**) 10 M/mL for HCT-116 and (**iv**) 10 K/mL, (**v**) 1 M/mL, (**vi**) 10 M/mL for MC3T3-E1.

To evaluate whether the prepared inks are suitable for cell encapsulation, MC3T3-E1 and HCT-116 were encapsulated, and viability was first assessed through the MTS assay. For both cell types (see Figure 9a,b), the results obtained after 1 day showed, as expected, a decrease in the absorbance value with decreasing cell density (10 M/mL, 1 M/mL, 10 K/mL). However, after 7 days, it is possible to observe a reduction in absorbance and, therefore, in cell viability for the density of 10 M/mL; on the other hand, as the cell density decreases, a progressive increase in absorbance is observed. This is probably due to the amount of material being insufficient to support the continuous growth of the higher cell number—in particular, HCT-116—but sufficient for a smaller number of cells (1 M/mL and 10 K/mL) to ensure their progressive proliferation. For MC3T3-E1, an analogous trend was registered; however, the MTS test showed lower absorbance than HCT-116 at the same cell density considered. This can be related to a higher metabolic activity and proliferation of HCT-116 than MC3T3-E1, which may explain the significant difference in the results obtained. However, for both, it can be observed that, after mixing, cell viability was not compromised. Finally, it can be observed how, for each cell density, at almost any time, the cells encapsulated in bioink A show slightly higher viability than those encapsulated in bioink B. This may be due to the different mechanical features of the two polymers, with B being less structured than A, which may not be able to support cell viability and growth.

To qualitatively confirm the MTS assay, the Live&Dead assay was performed. The results are shown in Figure 9c–f. As can be seen, most of the cells maintained their viability and, only sporadically, the presence of non-viable cells was found. Finally, the cells were successfully encapsulated and homogeneously distributed in all constructs and the encapsulation and crosslinking did not result in harmful effects on cell viability.

## 4. Conclusions

We have demonstrated the design and preparation of a gellan gum-based hydrogel that can be applied as a bioink to fabricate cell-laden structures using an extrusion-based 3D printer. In fact, while GG-EDA, due to the ionic interactions described, provides dispersions that are too viscous at the concentrations used in printing, the mixing with PEG-aldehyde and the consequent formation of a dynamic linkage allows the modulation of its viscoelastic properties and improvement of the extrusion process. At the same time, the removal of PEG in physiological fluids allows the restoration of the properties of GG-EDA. Therefore, we demonstrated that the physicochemical, biological and rheological properties of GG-EDA-PEG derivatives are suitable for biomedical applications. Both the inks and the bioinks generally showed a pseudoplastic trend and good recovery of the initial viscoelastic properties. 

The cell compatibility studies have shown how the preparation and mixing process of the bioinks is compatible with the cell viability and therefore both the samples used are cytocompatible. 

Moreover, in this work, we investigated the effect of cell type (HCT-116 and MC3T3-E1), cell densities (10 K/mL, 1 M/mL and 10 M/mL) and ionotropic crosslinking on rheological properties. Although different results were obtained depending on the structure of the bioink and the type of cell, the rheological analyses confirmed that cell density was not neglected when designing a new bioink.

## Data Availability

Not applicable.
